# Internet use May be associated with the severity of headache in migraine patients: results from a Hungarian tertiary headache center

**DOI:** 10.1186/s12889-025-22255-9

**Published:** 2025-03-18

**Authors:** Anett Csáti, Frida Horváth, Délia Szok, Antal Tibold, Ildiko Radvanyi, Eva Fejes, János Tajti, Gergely Fehér

**Affiliations:** 1 Department of Neurology, Albert Szent-Györgyi Health Centre, University of Szeged, Szeged, Hungary; 2 Albert Szent-Györgyi Medical School, University of Szeged, Szeged, Hungary; 3 Centre for Occupational Medicine, Medical School, University of Pécs, Pécs, 7627 Hungary; 4 Hospital of Komló, Komló, Hungary

**Keywords:** Internet use, Migraine patients, Severity of migraine attack

## Abstract

**Background:**

Migraine as a prevalent primary headache disorder affects mainly the young population. Migraines worsen the quality of life and are responsible for the main cause of disability worldwide. Apart from the well-known lifestyle factors, the spread of digitalization seems to influence the frequency as well as the severity of migraine attacks, however clinical studies are still lacking. Here we present a prospective single-center cross-sectional study focusing on the possible negative effects of being online and problematic internet use on the severity of headache during migraine attacks taking many covariates into account.

**Methods:**

Migraine patients were recruited from the Headache Outpatient Clinic of the Department of Neurology, University of Szeged, Szeged, Hungary. Socio-demographic data, medical history of migraine, severity of migraine attacks and details of online activities were recorded as well as online questionnaires focusing on internet addiction.

**Results:**

A total of 192 patients (*n* = 166 female; *n* = 26 male) completed the online questionnaire package. After the setup of a logistic regression among socio-demographic data age > 45 years (odds ratio, OR = 1.101), being married (OR = 1.584), number of children > 2 (OR = 1.227), secondary employment (OR = 2.349), while related to being online only nighttime internet use (OR = 1.330) was significantly associated with the severity of migraine attacks.

**Conclusion:**

This study is among the first clinical studies focusing on the unfavorable effects of online activities on the severity of headache in migraine patients. Clinicians should be aware the negative effect of being online as a potential non-pharmacological aspect of migraine treatment.

## Background

Migraine is a frequent multifactorial neurovascular disorder with predominance in females with high personal and socioeconomic impact [1]. Based on the results of the Global Burden of Disease (GBD) Study 2021 almost 1.16 billion individuals suffered from migraine worldwide [2]. Migraine caused approximately 43.4 million global years lived with disability (YLDs) [2]. Global prevalence of migraine was 15.2% (females 18.9%, males 11.4%) [3]. The results GBD 2021 demonstrated that migraine was second among the world’s causes of disability in both genders with age range 20–59 years, and was first among young female migraine sufferers [2]. For detailed measurement of disability, neuropsychological tests can be used [4]. Epidemiological studies resulted in the obvious statement that migraine had a negative impact on health-related quality of life [5–7].

Apart from the well-known lifestyle factors, such as stress, malnutrition or poor posture, the spread of digitalization as a new form of addiction in the 21st century seems to affect the frequency as well as the severity of migraine attacks [8, 9]. Out of digital technologies first the excessive computer and video game use were examined in association with their impact on headache. More than 4 h daily use of electronic devices among adolescents in Brazil, and more than 8 h per day computer use among information technology professionals in China were trigger factors for contributing to headache [10, 11]. According to systematic reviews and meta-analyses of cross-sectional clinical studies, the risk of headache was increased among mobile phone users, and in case of longer and more frequent daily calls [12, 13]. Based on a scoping review headache is also associated with problematic social media usage (see below) in children and adolescents [14].

The internet has become an integral part of our lives, serving as a primary source of information related to work, education, leisure, and communication. While a healthy, controlled use of the web has an undeniably positive impact on our daily lives, excessive, maladaptive use can have a detrimental effect on an individual’s mental and physical health. The problematic internet use (PIU) or internet addiction (IA) refers to excessive online activities that result in significant functional impairment and/or distress [15, 16]. The term of IA was suggested by Ivan Goldberg in 1995 [17]. It is still a matter of debate, whether it is a novelty in the family of addictions or a new manifestation of an existing psychiatric disease. It is also not entirely clear whether all digital addictions can be considered as a single entity or individual subtypes (general internet addiction, mobile addiction, social media addiction, etc.) should be considered as different pathologies [18].

As IA is not listed in the Diagnostic and Statistical Manual of Mental Disorders, Fifth Edition, its definition is various [19]. IA is usually defined as “a problematic, compulsive use of the internet, resulting in significant impairment in an individual’s function in various life domains over a prolonged period of time” [20]. IA seems to have several risk factors, such as male gender, younger age, low socioeconomic status and impaired family relationships, but results are controversial, and may vary on age and goals on internet use [15, 21, 22]. The prevalence of IA can vary between 0.8 and 26.7% depending on geographical areas and age, but it is more common among adolescents and Far-East groups [23]. The pooled prevalence of IA was 7.02% in a 7-60-year-old population [24]. Our recent studies showed that it can be as high as 19.1% in high-school students [22] and 5.2% in an adult Hungarian population [25]. IA shows connection to mental and somatic disorders, such as anxiety, depression, sleep disturbances, attention deficit hyperactivity disorder (ADHD), malnutrition, hypertension, diabetes, cardiovascular diseases or musculoskeletal disorders [26–29].

The connection between IA and headache disorders is rarely studied. The vast majority of epidemiological studies showed a possible association between the presence and severity of migraine among problematic internet users, but clinical studies are missing [22]. The Outpatient Headache Clinic Headache of the University of Szeged is one of the oldest and scientifically active tertiary centers covering nearly a million inhabitants [30]. As clinical studies are missing, focusing on the association of migraine and problematic usage of the internet, the aims of this study were designed (1) to determine the prevalence of IA among adult migraine patients attending to our tertiary headache clinic, (2) to identify the potential associative factors for the severity of migraine attacks, and (3) to analyze the possible connection between IA and the severity of migraine.

## Methods

### Study design and participants

This study was carried out from April to July 2022 at the tertiary center Headache Outpatient Clinic of the Department of Neurology, Albert Szent-Györgyi Health Centre, University of Szeged, Szeged, Hungary [30]. It was an online questionnaire-based cross-sectional single-center convenience (non-random) sampling trial among attendants of the headache clinic. The study protocol and documentation were approved by the Ethical Committee of the University of Szeged (ethical permission number: 192/2015). Participation was anonymous and voluntary.

Inclusion criteria were the established diagnosis of migraine with or without aura based on the ICHD-3 diagnostic criteria [31] with the use of the validated Hungarian version of the ID-migraine questionnaire [32] and the age of above 18 years. Migraine was diagnosed by neurologists, specialized to headache disorders. Demographic data included gender, age, marital status, number of children, qualification, work schedule and secondary employment. Included lifestyle factors were tobacco use, regular alcohol and coffee intake. Medical conditions taken into account were the history of seizures, malignancies, diabetes, hypertension, musculoskeletal (cervical, lumbar) pain and psychiatric (depression, anxiety, attention deficit, impulse-control disorder, insomnia, schizophrenia) disorders. The age of onset of the headache disorder, the number of monthly migraine days, the duration of the migraine attacks, lateralization and location, quality and intensity of the headache, the presence of aura, concomitant clinical signs (such as nausea or photophobia), and the provoking or ceasing factors were investigated. The severity of migraine pain was established by using 11-points Numeric Rating Scale (NRS), where 1–4 points present mild, 5–6 points moderate, and above 7-point severe headache.

IA was examined with the use of the Problematic Internet Use Questionnaire (PIU-Q), which is a validated self-reported scale with good reliability [33]. The questionnaire contains 18 items, each scored on a 5-point Likert-type scale ranging from 1 (never) to 5 (always). A total score exceeding 41 points suggests IA [26, 33]. Daily time intervals and goals of internet use were also recorded. Based on the PIU-Q results, participants were categorized as (1) problematic internet users (IA) or (2) normal internet users (not addicted to the internet) (Chronbach alpha’s 0.909).

### Statistical analysis

For a study evaluating the association between internet addiction and migraine severity, with a moderate effect size (Cohen’s f = 0.25), 80% power, and a significance level of 0.05, the minimum required sample size was 128 participants.

Data were evaluated as means ± standard deviation (SD) by Student’s t-test, the chi-square test and the Pearson’s Rank-Order Correlation. To clarify the role of different parameters as independent risk factors for the severity of migraine, logistic regression analysis was carried out including all the examined parameters (sociodemographic data, trigger factors, concomitant diseases and internet use). For all odds ratios (OR), an exact confidence interval (CI) of 95% was constructed. Data analysis was performed using SPSS (version 22.0, IBM, New York, NY, USA).

## Results

### Sociodemographic characteristics

Overall 315 questionnaires were successfully delivered online, of which 192 responses (13.5% male and 86.5% female) were received (response rate of 60.9%). Due to the age distribution of study participants, 35.4% were 18–35 years old and 51.1% were between 36 and 55 years of age, the rest were older than 55 years. The majority (74%) of the study population was married or lived in a relationship. The ratio of the patients who have two or more children was 38.6%. Regarding the education level of the study population 56.8% graduated in high school and 40.6% reached higher education level. Regarding the work schedule, 63.0% of the subjects worked in a single shift schedule. 12% of the study subjects had a second job. Regarding the substance use 16.7% of the study population were regular smokers, 25.5% alcohol users and 64.6% drank 1 or 2 coffee per day (Table 1).

**Table 1:** Characteristics of the study population

	Participants
	*N* = 192 (%)
**Gender**	
male	26 (13.5)
female	166 (86.5)
**Age**	
18–25 years	24 (12.5)
26–35 years	44 (22.9)
36–45 years	47 (24.5)
46–55 years	51 (26.6)
56–65 years	19 (9.9)
66–75 years	7 (3.6)
**Marital status**	
single	28 (14.6)
divorced/widow	22 (11.4)
married	86 (44.8)
relationship	56 (29.2)
**Number of children**	
have no child	71 (36.9)
1 child	47 (24.5)
2 children	57 (29.7)
more than 3 children	17 (8.9)
**Graduation**	
elementary	5 (2.6)
secondary education	109 (56.8)
higher education	78 (40.6)
**Work schedule**	
single shift	121 (63.0)
two shifts	26 (13.6)
continuous	45 (23.4)
**Another part-time job**	
no	169 (88.0)
have secondary employment	23 (12.0)

### Characteristics of migraine

In the study population 65.1% had migraine without aura and 34.9% had migraine with aura. The age onset of migraine was in childhood and adolescence (6–19 years) in 52.1% of the study population and in young adulthood (between 20 and 30 years) was 26.6% of them. Regarding the severity of migraine attacks 80.2% of the patients reported severe (NRS > 7/10) headache. Due to the duration of migraine attacks the majority (37.0%) of the study patients reported 3–12 h-long painful episodes (Table 2).

**Table 2:** Migraine characteristics of the study population

	*N* = 192	%
**Age of onset of migraine**		
6–14 years	57	29.7
15–19 years	43	22.4
20–30 years	51	26.6
31–40 years	25	13.0
41–50 years	12	6.3
more than 50 years	4	2.0
**Severity of migraine pain**		
mild	9	4.7
moderate	29	15.1
severe	154	80.2
**Migraine days per month**	6.4±5.3	
**Duration of symptoms**		
less than 3 h	31	16.1
3–12 h	71	37.0
12–24 h	37	19.3
24–48 h	22	11.5
48–72 h	21	10.9
more than 72 h	10	5.2
**Type of migraine**		
Migraine without aura	125	65.1
Migraine with aura	67	34.9
- visual aura	40/67	59.7
- motor aura	13/67	19.4
- sensory aura	3/67	4.5
- retinal aura	11/67	16.4

### Internet use

Regarding the daily internet use the most (26.5%) of the study participants spent an average of 2 h online. Due to 3 h-periods of the day the preferred time being online (based on multiple answers) was between 6:00 p.m. and 9:00 p.m. of the majority of the patients (59.4%). Considering the goal of internet use based on multiple answers, the highest ratio of the subjects used the internet for learning or working (57.3%), then 33.9% of them for watching movies and 33.3% of them for video streaming. Online gaming was detected in 8.3% of the study population (Table 3).

**Table 3:** Internet use in the study population

	Participants	
	*N* = 192 (%)	
**Daily internet use (approximately)**		
< 1 h		22 (11.5)
1 h		28 (14.6)
2 h		51 (26.5)
3 h		33 (17.2)
4 h		16 (8.3)
5 h		8 (4.2)
6 h		14 (7.3)
> 6 h		20 (10.4)
**Daily time interval of internet use (multiply answer)**		
between 12:00 a.m. and 3:00 a.m.		5 (2.6)
between 3:00 a.m. and 6:00 a.m.		5 (2.6)
between 6:00 a.m. and 9:00 a.m.		61 (31.8)
between 9:00 a.m. and 12:00 a.m.		77 (40.1)
between 12:00 a.m. and 3:00 p.m.		68 (35.4)
between 3:00 p.m. and 6:00 p.m.		68 (35.4)
between 6:00 p.m. and 9:00 p.m.		114 (59.4)
between 9:00 p.m. and 12:00 p.m.		36 (18.8)
**Goal of internet use** **(multiply answer)**		
learning/working		110 (57.3)
chat		30 (15.6)
movies		65 (33.9)
music		56 (29.2)
social media		48 (25.0)
video streaming		64 (33.3)
online gaming		16 (8.3)
other		32 (16.7)

### Problematic internet use

Based on the definition, 4.2% of the study migraine population belongs to the PIU category. The point of view of the age distribution of PIU subjects 50% of them was between 26 and 35 years old. Regarding the severity of migraine attacks 6 out of the all 8 PIU patients rank in the severe headache intensity group **(**Table 4**)**.

**Table 4:** Problematic internet use of the study population in aspects of the gender, age and the severity of migraine attacks

	Participants		Severity of migraine headache		
		Total	Mild	Moderate	Severe
		*N* = 192 (%)	*N* = 9 (%)	*N* = 29 (%)	*N* = 154 (%)
**Problematic internet use**		**8 (4.2)**	**0**	**2 (6.9)**	**6 (3.9)**
**Gender**					
Male		5 (62.5)	0	1 (50.0)	4 (66.7)
Female		3 (37.5)	0	1 (50.0)	2 (33.3)
**Age (years)**					
18–25		1 (12.5)	0	0	1 (16.7)
26–35		4 (50)	0	1 (50.0)	3 (50.0)
36–45		1 (12.5)	0	0	1 (16.7)
46–55		1 (12.5)	0	0	1 (16.7)
56–65		0 (0)	0	0	0
66–75		1 (12.5)	0	1 (50.0)	0

### Probable risk factors for the severity of migraine attacks

By univariate analysis including all factors, out of the sociodemographic data age 46–65 years (46–55 years *p* = 0.014, 56–65 years *p* = 0.025), being married (*p* = 0.045), number of children (*n* = 2 *p* = 0.012, *n* = 3 or more *p* = 0.035) and secondary employment (*p* = 0.022) were associated with the severity of migraine attacks **(**Table 5**)**. Due to internet-related data nighttime (from 9:00 p.m. to 12:00 p.m.) internet use (*p* = 0.034), and playing online games (*p* = 0.038) also showed significant connection with the severity of migraine attacks **(**Table 6**)**. Out of lifestyle factors multiple coffee intake (*p* = 0.041) was associated with more severe migraine attacks. Based on the significant association due to univariate analysis, multivariate analysis including the above mentioned parameters showed that being married (*p* = 0.031, OR = 1.141 CI 95% 1.030–1.247), number of children > 2 (*p* = 0.021, OR = 1.227 CI 95% 1.197–1.322), secondary employment (*p* = 0.029, OR = 1.245 CI 95% 1.069–1.471), and nighttime (from 9:00 p.m. to 12:00 p.m.) internet use (*p* = 0.018, OR = 1.701 CI 95% 1.641–1.819) revealed significant relation to the severity of migraine attacks.

**Table 5:** Sociodemographic risk factors for the severity of migraine attacks. (*p < 0.05)

	Severity of migraine headache					*P* value
	Mild	Moderate		Severe		-
	N = 9 (%)		N = 29 (%)		N = 154 (%)	-
**Gender**						
male	1 (11.1)		3 (10.3)		22 (14.3)	0.103
female	8 (88.9)		26 (89.7)		132 (85.7)	0.751
**Age**						
18–25 years	2 (22.2)		3 (10.3)		19 (12.3)	0.605
26–35 years	2 (22.2)		6 (20.6)		36 (23.4)	0.790
36–45 years	5 (55.6)		6 (20.6)		36 (23.4)	0.662
46–55 years*	*0 (0)*		*8 (27.8)*		*43 (27.9)*	*0.014*
56–65 years*	*0 (0)*		*2 (6.9)*		*17 (11.0)*	*0.025*
66–75 years	0 (0)		4 (13.8)		3 (2.0)	0.975
**Marital status**						
single	4 (44.4)		1 (3.4)		23 (14.9)	0.317
divorced/widow	2 (22.2)		3 (10.3)		17 (11.0)	0.269
married*	*0 (0)*		*13 (44.9)*		*73 (47.4)*	*0.045*
relationship	3 (33.4)		12 (41.4)		41 (26.7)	0.255
**Number of children**						
have no child	7 (77.8)		8 (27.8)		56 (36.4)	0.475
1 child	1 (11.1)		8 (27.8)		38 (24.7)	0.658
2 children*	*1 (11.1)*		*8 (27.8)*		*48 (31.2)*	*0.012*
more than 3 children*	*0 (0)*		*5 (16.6)*		*12 (7.7)*	*0.035*
**Graduation**						
elementary	0 (0)		1 (3.4)		4 (2.6)	0.251
secondary education	5 (55.6)		17 (58.6)		87 (56.5)	0.610
higher education	4 (44.4)		11 (38.0)		63 (40.9)	0.600
**Work schedule**						
single shift	6 (66.7)		18 (62.1)		97 (63.0)	0.797
two shifts	2 (22.2)		4 (13.8)		20 (12.9)	0.131
continuous*	*1 (11.1)*		*7 (24.1)*		*37 (24.1)*	*0.049*
**Another part-time job**						
no	9 (100)		25 (86.2)		135 (87.7)	0.567
have secondary employment*	*0 (0)*		*4 (13.8)*		*19 (12.3)*	*0.022*

**Table 6:** Internet use in the study population in aspects of the severity of migraine attacks. (*p < 0.05)

Participants		Severity of migraine headache			*P* value	
Total	Mild		Moderate	Severe		
*N* = 192	*N* = 9 (%)		*N* = 29 (%)	*N* = 154 (%)		
**Daily internet use (approximately)**						
< 1 h	0 (0)		4 (13.8)	18 (11.7)	0.306	
1 h	1 (11.1)		3 (10.3)	24 (15.6)	0.271	
2 h	1 (11.1)		8 (27.6)	42 (27.3)	0.273	
3 h	1 (11.1)		4 (13.8)	28 (18.2)	0.265	
4 h	2 (22.2)		3 (10.3)	11 (7.1)	0.623	
5 h	1 (11.1)		0 (0)	7 (4.5)	0.653	
6 h	2 (22.2)		2 (6.9)	10 (6.5)	0.671	
> 6 h	1 (11.1)		5 (17.3)	14 (9.1)	0.516	
**Daily time interval of internet use (multiply answer)**						
between 0:00 a.m. and 3:00 a.m.	0 (0)		0 (0)	5 (3.2)	0.304	
between 3:00 a.m. and 6:00 a.m.	0 (0)		2 (6.9)	3 (2.0)	0.751	
between 6:00 a.m. and 9:00 a.m.*	*1 (11.1)*		*9 (30.9)*	*51 (33.1)*	*0.019*	
between 9:00 a.m. and 12:00 a.m.	3 (33.4)		17 (58.6)	57 (37.0)	0.194	
between 12:00 a.m. and 3:00 p.m.	6 (66.7)		7 (24.1)	55 (35.2)	0.304	
between 3:00 p.m. and 6:00 p.m.	4 (44.4)		9 (30.9)	55 (35.2)	0.652	
between 6:00 p.m. and 9:00 p.m.	7 (77.8)		15 (51.7)	92 (59.7)	0.147	
between 9:00 p.m. and 12:00 p.m.*	*1 (11.1)*		*3 (10.3)*	*32 (20.8)*	*0.034*	
**Goal of internet use** **(multiply answer)**						
learning/working	6 (66.7)		19 (65.5)	85 (55.2)	0.503	
chat	8 (88.9)		16 (55.2)	6 (4.0)	0.577	
movies	4 (44.4)		6 (20.6)	55 (35.2)	0.460	
music	3 (33.4)		5 (16.6)	48 (31.2)	0.664	
social media	7 (77.8)		14 (48.3)	27 (17.5)	0.629	
video streaming	4 (44.4)		13 (44.8)	47 (30.5)	0.202	
online gaming*	*0 (0)*		*3 (10.3)*	*13 (8.4)*	*0.038*	
other	1 (11.1)		5 (16.6)	26 (16.8)	0.809	

## Discussion

Many factors have been identified as influencing migraine so far [34], but recent studies also raised the role of digital addictions. Many aspects of digital addictions were evaluated among different populations in the last three decades. Based on the recent literature data regarding the effect of IA clinical studies were conducted on various healthy subjects and not on disease-specific populations [17, 20, 25, 26, 35–40]. Regarding headache-related issues correlated to IA are underinvestigated and the results are contradictory comparing to their clinical importance. Available headache-related IA data are sparse and they mainly focus on the childhood and adolescence periods.

Among conventional factors, being older than 45 years was associated with more severe migraine attacks. This may be attributed to the higher proportion of women (and middle-aged women) due to peri- or postmenopausal hormonal changes [41]. This is slightly controversial to previous studies carried out in the beginning of the 21st century, but recent publications showed the increased rate of severe migraine attacks among those above 50, apart from hormonal background, the role of obesity can also be a significant predecessor [41, 42]. However, age was not a significant predictor in a multivariate analysis.

Being married was also associated with the severity of migraine in both uni- and multivariate analysis. This has not been published before and we have no clear explanation to our findings. We hypothesize the potential role of stress, which is closely related to the severity of migraine attacks, and the presence of sexual dysfunction causes more problems for married people compared to those living non-conventional relationships, but it is more speculative than scientific explanation [43].

Having more than two children also increased the possibility of worsening migraine attacks as well as having secondary employment in both uni- and multivariate analysis. Since the vast majority of the study consists of females, these results may also be due to increased stress and accelerated life as according to virtually all epidemiological surveys, women do most of the housework in addition to their work [44].

Despite the efficacy of caffeine intake in the treatment of acute attacks, increased daily intake may precede the severity of migraine. Our results are comparable to a recent study from the US, which showed a strong association between caffeine intake and severity of headaches [45]. The explanation is not entirely clear, the role of dehydration cannot be ruled out and chronic activation of the adenosine receptor (caffeine is structurally similar to adenosine) can result in dependence and headaches [45, 46]. Furthermore, caffeine can affect premenopausal hormone levels, which are strongly related to the development of migraine [45, 47].

Apart from the well-known trigger factors of migraine, recent studies raised the possibility of digital device use and digital addictions as novel factors, with controversial results [48–51]. These studies were epidemiological in nature, self-reported scales were included. While in a cross-sectional study among adolescent students no significant connection was found between the different digital abuses and primary headache disorders [48], a Turkish clinical study showed a 3.7% prevalence of IA in migraine adolescent population [49]. In our clinical study the rate of IA was 4.2%, which is comparable to recent studies carried out among adults in Hungary [44, 45]. According to a cross-sectional study conducted among Brazilian university students migraine with aura was significantly associated with IA and positive correlation was found between the severity of IA and the severity of headache [17]. On the other hand some aspects of internet use such as online gaming, nighttime internet use and smartphone use were associated with duration and frequency of migraine attacks and increased the administration of acute anti-migraine drugs [50, 51].

There are two explanations how being online (screen time) can participate in the development of migraine. The luminosity or frequency of screen band light may directly trigger the migraine cascade, on the other hand, increasing screen time exposure may reduce the threshold for a migraine attack, but these are more speculative than scientific explanations [52, 53].

IA was not included in the current DSM and ICD classifications but online gaming was labeled as a mental disorder [19]. Long hours of online gaming were associated with addiction and several mental conditions as well as with the presence of musculoskeletal pain and other pain syndromes [54]. Playing online games can trigger headaches based on a very recent study from Japan [55]. Abnormal postures, malnutrition and dehydration can be in the background of developing headaches in this population [56]. However, online gaming was not a predictor in a multivariate analysis.

On the other hand, nighttime internet use was associated with severe migraines in both uni- and multivariate analysis. Nighttime internet use can be the predecessor of addiction (with subsequent mental issues) and is associated with sleep deprivation or insomnia, all of the previous factors are well-known in the development and worsening of migraine [57]. Furthermore, the screen radiation of different digital devices can also trigger headaches [58].

In conclusion, parallel to the widespread use of digital devices, we have to face new factors of devastating pain syndromes occurring in medical practice. It is advisable to draw patients’ attention to potential risk factors avoiding the worsening of migraine. Besides patient education, cognitive behavioural therapy, and biopsychosocial approach can be favorable during the non-pharmacological management of migraine [34, 59].

Although our study is the first clinical research on the topic, it has some limitations. Due to the nature of the study, it was not representative neither in the general nor in the headache population and the applied methodology increased the risk of bias. The severity of migraine was evaluated by Numeric Rating Scale as Migraine Diability Scale and other widely used instruments are not validated in Hungarian language. The definition of IA is still a matter of debate due to the lack of proper studies and guidelines and influencing factors of migraine, goals of internet use and IA were defined on self-administered questionnaires.

## Data Availability

The datasets used and/or analyzed during the current study are available from the corresponding author on reasonable request.
